# Duodenal Tube Feeding: An Alternative Approach for Effectively Promoting Weight Gain in Children with Gastroesophageal Reflux and Congenital Heart Disease

**DOI:** 10.1155/2013/181604

**Published:** 2013-04-09

**Authors:** Seiko Kuwata, Yoichi Iwamoto, Hirotaka Ishido, Mio Taketadu, Masanori Tamura, Hideaki Senzaki

**Affiliations:** ^1^Department of Pediatrics and Pediatric Cardiology, Saitama Medical University, Saitama, Japan; ^2^Department of Pediatric Cardiology, Saitama Medical Center, Saitama Medical University, Staff Office Building 101, 1985 Kamoda, Kawagoe, Saitama 350-1981, Japan

## Abstract

We tested whether duodenal tube feeding effectively improves the clinical symptoms and body weight gain in children with congenital heart disease (CHD) and gastroesophageal reflux (GER). In the retrospective analysis of 17 consecutive children with CHD who were treated with duodenal tube feeding for symptomatic GER, we found that clinical symptoms of persistent emesis or respiratory wheezing after feeding disappeared with duodenal tube feeding in all patients. Duodenal tube feeding facilitated a stable nutritional supply, resulting in marked improvement of weight gain from 6 to 21 g/day (*P* < .0001). In a patient with trisomy 21 and persistent pulmonary hypertension after the closure of a ventricular septal defect, duodenal tube feeding ameliorated pulmonary hypertension, as evidenced by the improvement of the pressure gradient of tricuspid regurgitation from 77 to 41 mm Hg. In 14 of the 17 patients, the duodenal tube was successfully removed, with the spontaneous improvement of GER (median duration of duodenal tube feeding: 7 months). In conclusion, duodenal tube feeding improves the weight gain of infants with GER who need treatment for CHD-associated heart failure. It also allows for the improvement of pulmonary hypertension.

## 1. Introduction

Body weight gain is important for the successful treatment for infants with heart failure associated with congenital heart disease (CHD). Gastroesophageal reflux (GER) is known to be relatively common in this condition and is occasionally an important cause of growth failure in affected patients [1]. It can also cause aspiration pneumonia and pulmonary arterial hypertension, thus potentially complicating the clinical course of heart failure [2]. Medical therapy with gastric acidity inhibitors, including histamine-2 receptor antagonists and proton pump inhibitors, is the first line of treatment; however, it is not always effective [3–6]. In such cases, antireflux surgical procedures are selected [7–10]. Another treatment option may be the administration of duodenal tube feeding, which is less invasive than surgical procedures and thus may be beneficial for this particular group of patients for whom invasive interventions with general anesthesia carry a risk for worsening heart failure. However, little information is available about the efficacy of duodenal tube feeding for infants with GER and heart failure associated with CHD. In this study, we reviewed our experience of duodenal tube feeding performed in 17 children with CHD-associated heart failure, focusing on its efficacy in terms of body weight gain. We also evaluated its effect on GER-induced pulmonary hypertension.

## 2. Methods

Seventeen consecutive infants and children with preoperative (*n* = 3) and postoperative (*n* = 14) CHD and heart failure who were treated with duodenal tube feeding were analyzed. These patients had episodes of frequent vomiting and/or wheezing after oral or tube feeding and therefore were suspected of having GER. They underwent gastrography, which showed a reflux of contrast medium from the stomach to the esophagus beyond the halfway point between these organs. After gastrography, a weighted duodenal tube (5 Fr) was inserted under fluoroscopic guidance using a guidewire within a tube to facilitate manipulation of the tube that was then advanced beyond the descending portion of the duodenum. The appropriate position of the tube was finally confirmed by injecting a small amount of contrast medium through the tube, which showed the jejunum directly. A gastric tube is routinely placed for medication but not for gastric acid drainage. Because our patients had no gastrointestinal tract obstruction, gastric tube drainage was not performed in order to avoid potential electrolyte disturbance. Medication for reducing acid levels was continued only for severe GER patients who showed a reflux of contrast medium up to the pharynx.

We compared the body weight gain averaged for 14 to 21 days before and after duodenal tube feeding in each patient. In 1 patient (trisomy 21) who showed persistent pulmonary hypertension after the closure of a ventricular septal defect, changes in the severity of pulmonary hypertension were assessed by measuring the Doppler flow velocity of tricuspid regurgitation (TR). 

## 3. Results 

**Figure 1:**
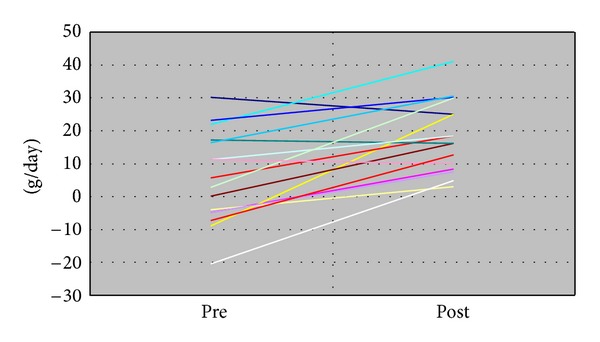
Changes in body weight gain per day before and after duodenal tube feeding.

Table 1 summarizes the characteristics of the studied patients. Of note, 13 patients had underlying conditions of chromosomal abnormalities (*n* = 10) or anomaly syndromes (*n* = 3). The patients' age at the time of the initiation of duodenal tube feeding ranged from 0 to 16 months, with a median of 2 months. No adverse events occurred during the insertion of the duodenal tube. In all patients, clinical symptoms of persistent emesis or respiratory wheezing after feeding disappeared after duodenal tube feeding. Duodenal tube feeding facilitated a stable nutritional supply, resulting in marked improvement of weight gain from 6 to 21 g/day (*P* < .0001, Figure 1). In the patient with trisomy 21 and persistent pulmonary hypertension after the closure of a ventricular septal defect, duodenal tube feeding ameliorated the pulmonary hypertension, as evidenced by the improved pressure gradient of TR from 77 to 41 mmHg.

In 14 of the 17 patients, the duodenal tube was successfully removed, with the spontaneous improvement of GER. The median duration of duodenal tube feeding was 7 months, ranging from 4 to 10 months. One patient who had single ventricular physiology complicated with asplenia syndrome underwent laparoscopic fundoplication after the initial cardiac surgery (the Blalock-Taussig shunt). In this particular patient, the procedure of fundoplication was difficult because of the unusual anatomical relation between the heart and the stomach, and the patient developed hypotension and cyanosis during the procedure. Another patient died of severe heart failure after cardiac surgery for the Ebstein anomaly. The remaining patient had trisomy 18 and continued duodenal tube feeding, considering the risk for both general anesthesia and antireflux surgery. 

Tube-related complications associated with duodenal tube feeding included accidental removal, obstruction, or damage; in most of the patients, these complications necessitated the replacement of the tube before the scheduled 3-month replacement. In addition, enterocolitis due to multiresistant *Staphylococcus aureus* (MRSA) was observed in 1 patient during the study period. 

## 4. Discussion

To the best of our knowledge, our study is the first to demonstrate that duodenal tube feeding is effective in promoting weight gain in infants and children with CHD and GER. 

In general, pharmacologic therapy with gastric acidity inhibitors, including histamine-2 receptor antagonists and proton pump inhibitors, together with maintaining an upright posture during feeding and the administration of thickened feedings, is the mainstay of GER treatment [3–6]. However, such medical therapy often fails to resolve the symptoms of GER in children [4–6]. In a particular group of infants with CHD, Weesner and Rosenthal [11] also reported very low success rates of medical therapy in resolving the infants' respiratory symptoms. In addition, the use of gastric acidity inhibitors was reported to be associated with an increased risk of acute gastroenteritis and community-acquired pneumonia in a multicenter, prospective study of children with GER [6].

In contrast, antireflux surgery for GER (i.e., the Nissen fundoplication) has been generally shown to result in a substantial improvement of reflux and the alleviation of its consequences in children without CHD [7–10]. Several studies have also reported that laparoscopic procedures can be safely performed even in infants with CHD, with careful monitoring of arterial carbon dioxide levels during insufflation [12, 13]. In addition, a recent study by Cribbs et al. [2] showed that the surgical repair of GER in their population of infants and children with severe CHD was safely performed and effectively promoted weight gain. However, they also reported 1 death among 112 procedures and 3 potentially lethal complications in the early postoperative period. These data evidence that pediatric cardiac anesthesia providers are essential for the safe performance of antireflux procedures in this population, with postoperative care administered in a dedicated cardiac intensive care unit, as suggested by the authors. Our patient with asplenia syndrome who underwent laparoscopic fundoplication indeed experienced unexpected hemodynamic instability during the procedure even under the care of our pediatric anesthesiologists. 

Compared with surgical procedures, insertion of a duodenal tube does not require general anesthesia or postoperative intensive care, which would be a major advantage of this approach. With the low procedural risk, duodenal tube feeding consistently ameliorated GER-related symptoms and resulted in a dramatic improvement of weight gain. In addition to being less invasive, duodenal tube feeding may have another merit in that it can be terminated upon the subsequent resolution of GER. There has been no clear information about whether GER associated with CHD can improve over time. In this sense, our study clearly demonstrated that most of our studied population outgrew reflux, and the duodenal tube was successfully removed within 10 months after the initiation. Because the timing of the follow-up gastrography to check for GER status was arbitrary in our patients, the periods of duodenal tube feeding could have been even shorter than the actual duration of 10 months. In the least, duodenal tube feeding can avoid antireflux surgery in symptomatic GER patients with CHD. Duodenal tube feeding may also be useful as a bridge to fundoplication particularly in preoperative CHD patients, as in our asplenia patient who underwent fundoplication after the temporal administration of duodenal tube feeding. Fundoplication may be more safely performed under stable hemodynamic conditions established after cardiac surgery. 

Our study also demonstrated the improvement of pulmonary hypertension by duodenal tube feeding in a trisomy 21 patient after a corrective surgery for CHD. This is consistent with our previous report on a Down syndrome infant without structural cardiac anomaly [14]. Down syndrome is known to be associated with an increased prevalence of GER. The syndrome is also known to pose a high risk for postoperative persistent pulmonary hypertension. The present case, together with our previous one, highlights the importance of a high index of suspicion for GER as a curable cause of pulmonary artery hypertension in this syndrome.

Finally, tube dislodgement, migration, diarrhea, and enterocolitis are known to occur occasionally with duodenal tube feeding. In fact, we experienced tube-related complications such as accidental removal, obstruction, and damage that necessitated unscheduled tube replacement in most patients. Education and training of both family and medical staff are essential for minimizing these events. In addition to these tube-related complications, 1 patient in our study experienced MRSA enterocolitis during duodenal tube feeding. Although a cause-effect relationship between duodenal tube feeding and the occurrence of MRSA enterocolitis was not clear, intestinal tract infection is a known potential important complication associated with duodenal tube feeding that may occur due to reduced protective effects of gastric acid. This fact also should be kept in mind during the application of duodenal tube feeding. 

## 5. Conclusions

Duodenal tube feeding is a less invasive method for promoting weight gain in symptomatic children with GER associated with CHD. It can be terminated upon the spontaneous resolution of GER with the patient's growth. Thus, duodenal tube feeding should be considered a useful alternative to surgery in CHD patients with GER. 

## Figures and Tables

**Table 1:** Patients' Characteristics.

Birth weight, g	2607 ± 321
Age at the time of diagnosis, months	4.7 ± 0.5
Body weight at the time of GER diagnosis	3667 ± 420
Types of congenital heart defects (*n*)	
Tetralogy of Fallot	4 (1)
Single ventricle	2 (1)
Atrioventricular septal defect	2 (1)
Coarctation of the aorta	1
Coarctation of the aorta with ventricular septal defect	1
Transposition of the great arteries	1
Double outlet right ventricle	2
Ventricular septal defect	1
The Ebstein anomaly	1
Patent ductus arteriosus	1
Other abnormalities (*n*)	
Trisomy 21	8 (2)
Trisomy 18	1
22q1-	2
Asplenia syndrome	2 (1)

Numbers in parentheses indicate postoperative patients.
